# Effect of larval swimming in the western North Pacific subtropical gyre on the recruitment success of the Japanese eel

**DOI:** 10.1371/journal.pone.0208704

**Published:** 2018-12-20

**Authors:** Yu-Lin K. Chang, Michael J. Miller, Katsumi Tsukamoto, Yasumasa Miyazawa

**Affiliations:** 1 Application Laboratory, Japan Agency for Marine-Earth Science and Technology, Yokohama, Japan; 2 Department of Marine Science and Resources, College of Bioresource Sciences, Nihon University, Fujisawa, Kanagawa, Japan; National Taiwan University, TAIWAN

## Abstract

The possible effect of directional larval swimming on the recruitment success of the Japanese eel, *Anguilla japonica*, was examined with a three-dimensional particle-tracking ocean circulation model using horizontal northwestward swimming and diel vertical migration (DVM). Four separate experiments included virtual larvae (v-larvae) movement from the spawning area over 290 days (total migration) and 160 days (stage A), from the STCC eddy region in 70 days (stage B), and from the origin of the Kuroshio in 60 days (stage C) to evaluate the effect of directional swimming and DVM compared to simple drifting. Passive or random swimming were not the most effective strategies for larvae dispersing from the spawning area because most v-larvae remained south of 20°N without entering the Kuroshio. Northwestward swimming resulted in wider dispersion and a better chance of successful recruitment, with v-larvae becoming widely distributed in the STCC eddy zone, arriving at the east coast of the Philippines (stage A), escaping the STCC eddy area and reaching the Kuroshio (stage B), and crossing the Kuroshio into the East China Sea shelf (stage C). DVM slightly shortened the migration period due to faster shallow layer ocean currents during nighttime. The NEC transported non-swimming v-larvae westward to the Kuroshio and occasionally northward into the Subtropical Countercurrent (STCC) area where eddies transported v-larvae westward into the Kuroshio, but less than with swimming. Directional swimming increased recruitment success, northwestward swimming was more effective than other directions, and a slower swimming speed was still better than no/random swimming in sensitivity tests. The present study demonstrated a first view of the possibility that Japanese eel larvae might be able to use a strategy of single-direction swimming to increase arrival at their recruitment areas.

## Introduction

Anguillid eels are widely distributed in the Indo-Pacific and North Atlantic regions with their juveniles living in estuarine and freshwater habitats and their reproduction and early life histories occurring in the open ocean [1, 2]. Their unusual catadromous life histories of spawning offshore and their larvae being transported by ocean currents towards their recruitment areas have contributed to a lack of understanding of what has caused the drastic declines of the northern hemisphere anguillid eel populations [3, 4]. The Japanese eel, *Anguilla japonica*, is one of the most important eel species for fisheries and aquaculture, and it has been listed as endangered on the IUCN red list [5]. Among the Northern Hemisphere species of eels that have all experienced declines, the Japanese eel seems to have shown the earliest recruitment declines that started in the 1970s [6]. After about 2010, its annual recruitment had decreased by as much as 90% compared to eel catches in the 1960s [5].

Although there are clear indications that species like the Japanese eel and Atlantic eels have experienced a variety of anthropogenic impacts such as drastic habitat reductions and overfishing [7–11], changes in the ocean-atmosphere system have also been evaluated for the potential to reduce recruitment of anguillid eels [12–15]. Changes in ocean productivity could cause increased early larval mortality due to a lack of food [11] or changes in spawning location or ocean current patterns could reduce successful larval transport [14, 16, 17].

**Fig 1:**
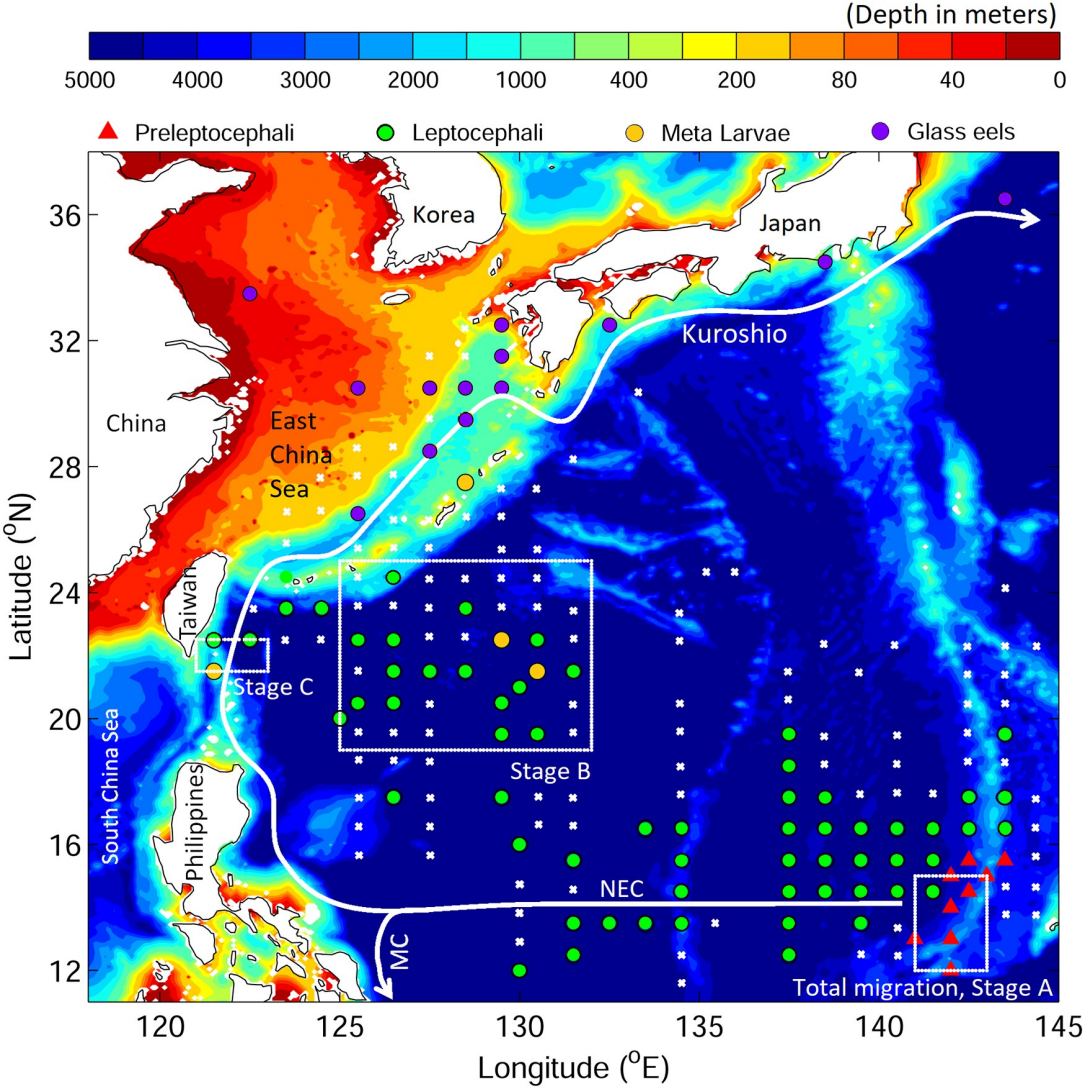
Ocean bathymetry in the western North Pacific Ocean and the observed eel larvae collection locations after Tsukamoto [28] and Shinoda et al. [29] pooled into 1° zones. Different eel stages are represented by different colors. Red triangles show preleptocephali, green dots leptocephali, gold dots metamorphosing larvae (meta), purple dots glass eels, and white crosses negative stations (no larvae caught). White boxes indicate release areas for stage A (in NEC), stage B (in STCC eddy area), and in stage C (in Kuroshio). Yellow lines show the 200 m depth contour. The collection locations of the larval stages are only suggestive of the overall distributions due to the limited sampling effort in most areas.

The Japanese eel appears to be especially vulnerable to changes in larval transport [16–21]. It is distributed across East Asia in areas that are adjacent to the western Pacific Ocean (Taiwan, China, Korea, Japan; Fig 1), but their spawning area is located far offshore along the West Mariana Ridge within the westward flowing North Equatorial Current (NEC) [22–24]. Their reproductive-stage eels (referred to as silver eels) migrate over a distance of thousands of kilometers to reach their spawning area where their eggs, newly hatched preleptocephali, and spawning condition adults have been collected [22, 23, 25–27]. The eel larvae, called leptocephali, are then transported from the spawning area by ocean currents, toward their growth habitats in the freshwater and estuarine habitats of East Asia [24, 28].

**Fig 2:**
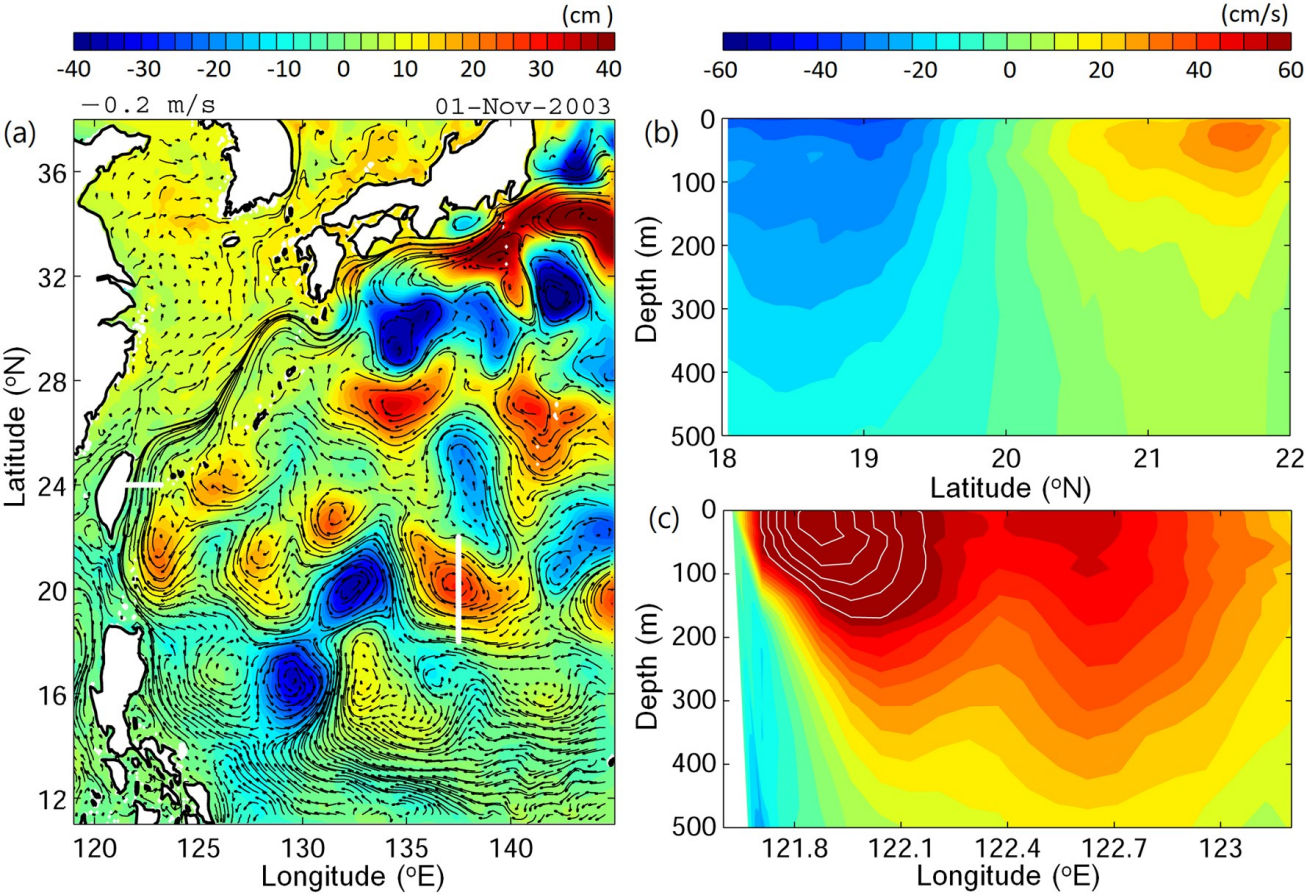
A snapshot of oceanic features in the western North Pacific Ocean in November 2003 showing sea surface height (color scales) and surface currents (arrows) (a) and cross-section (white lines in (a)) vertical profiles of an eddy (zonal velocity) (b) and across the Kuroshio (meridional velocity) (c). Positive values are eastward and northward in (b) and (c), respectively. In (c), the values exceeding the color bar are contoured, with a contour interval of 10 cm/s.

However, the ocean currents between the spawning area and growth habitats are highly variable (Fig 2a). The NEC is a westward current located mainly between 8−16°N, which then bifurcates into the northward flow (the Kuroshio) and southward flow (the Mindanao Current) [30, 31]. The STCC is located north of the NEC from 17−27°N and is a weak (~2 cm s^−1^) eastward flow that generates many mesoscale eddies with sizes of 150−300 km [32, 33]. STCC eddies have mean rotation speeds (U) of 20−30 cm/s (Fig 2b) and generally propagate westward at speeds (C) of 5−10 cm/s [34–36]. STCC eddies are nonlinear (nonlinearity is defined by the ratio of U to C, if nonlinear, U/C >1), which indicates eddy rotation is greater than its propagation, therefore, inferring a high potential of trapping material within the eddy interior [37, 38]. The westward propagating eddies eventually merge with the Kuroshio [35, 39]. The Kuroshio is the western boundary current of the North Pacific Ocean that flows along the east coast of the Philippines and Taiwan before entering the East China Sea and passing near the south coast of Japan as it turns offshore to the east and enters the Kuroshio Extension [40]. The Kuroshio has a strong speed of 1−2 m/s, a width of about 100 km, and its depth can penetrate to 600−1000 m (Fig 2c) [41, 42].

It has recently been pointed out that there are no apparent physical oceanographic processes that are able to passively transport large numbers of leptocephali across the main parts of strong western boundary currents such as the Kuroshio or Gulf Stream [43]. Some larvae could be moved across by eddies and rings cast off by large meanders in the currents, but these are not regular enough in space and time to move the whole population of larvae across the currents each year. So it appears that swimming would be required to cross or detrain from these powerful currents [43], and some modelling studies have shown that few larvae succeed to cross over to their recruitment habitats without swimming, or swimming with the currents [20, 44]. In subtropical gyres with complex circluation that can retain larvae [45], active swimming might also facilate moving out of the gyre towards recruitment areas [43], and some larval transport modelling studies support this possibility [20, 46].

Therefore to better understand the potential role of ocean current variability in causing recruitment reductions, it is important to begin to understand what effects active swimming may have on successful recruitment and if this might also be able to mitigate against ocean current changes farther offshore. The Japanese eel is a good model species for examining the possible effects of swimming because its oceanic life history has been intensively studied in recent years [23, 25, 29, 47]. Their larvae have been observed in the western Pacific (Fig 1) [28, 29], and the collections of their eggs, preleptocephali, and spawning-condition adults along the southern West Mariana Ridge seamount chainprovide information about specific spawning sites [23, 25–27]. The spawning latitudes are within the NEC, so leptocephali get transported westward and some have been collected in the STCC region where there are many mesoscale eddies [29]. Some metamorphosing leptocephali have been collected offshore or south of Taiwan and glass eels have been historically collected in the East China Sea (Fig 1), including in the Kuroshio in November or early December [48]. Metamorphosing larvae have also recently been collected in the STCC eddy region east of Taiwan [49]. These larval distributions can be used to infer the potential migration routes and dispersal patterns of Japanese eel larvae, but there has been a lack of sampling in the western NEC region where larvae must be present each year, but have not been collected in most areas.

Numerical modeling methods, therefore, have been used to simulate the potential migration paths of Japanese eel larvae [18–21], and the simulated larvae have sometimes been given behavioral characteristics. Some eel larvae show diel vertical migration (DVM) behavior by remaining in upper surface waters at night and then moving to deeper depths during the daytime [50]. DVM was often considered in the simulations by using either a two-layer structure [18, 19] or an age-dependent method [20]. In contrast, horizontal swimming was often ignored [18, 19, 21]. Although the swimming speed of Japanese eel larvae in the laboratory was relatively slow in response to changes in lighting conditions (3.6 ± 2.7 cm/s [51]) in comparison to major ocean features in western Pacific (10−100 cm/s), a recent study suggested that excluding swimming behavior could underestimate the dispersal of eel larvae migration, and the simulated migration duration from the spawning area to East Asia was close to actual estimates when an active swimming component was included [20]. Although including a swimming component might have been the better approach for simulating eel larvae migration, their results still failed to simulate actual arrival into the inner shelf of the East China Sea where the glass eels were observed. Instead, virtual eel larvae (v-larvae) were mostly transported by the Kuroshio and went downstream to the area south of Japan.

Swimming by late-stage larvae seems to be required in some cases, but because this has not been extensively investigated, it remains unclear if directional swimming by eel larvae is useful for moving over thousands of kilometers from the spawning area towards their growth habitats. The objective of the present study was to determine if directional swimming by Japanese eel larvae in the western North Pacific can increase the recruitment success to East Asia based on a particle tracking method. The roles of DVM and horizontal directional swimming were examined. The simulation of the larval migration towards East Asia was split into 4 geographic or time-period scenarios to assist in understanding the impact of major oceanic features on eel larvae migration. We examined the movement of larvae from the spawning area over 290 days (total migration) and 160 days (stage A), the movement of larvae from the STCC eddy region (stage B), and of larvae at the origin of the Kuroshio (stage C), to evaluate the effect of directional swimming and DVM compared to simple drifting on the success of the overall migration, the early migration, the ability to get out of the eddy region, and to cross out of the Kuroshio to reach recruitment areas.

## Data and methods

### Ocean reanalysis data: JCOPE2

The data-assimilative ocean circulation model known as the Japan Coastal Ocean Predictability Experiment 2 (JCOPE2) provided the three-dimensional currents and hydrological fields that were used for particle tracking in the present study. JCOPE2 was constructed from the Princeton Ocean Model with a generalized coordinate system [52]. The model domain of JCOPE2 encompasses the western North Pacific (10.5–62°N and 108–180°E), with a horizontal resolution of 1/12° (8–9 km) and 46 vertical layers. The lateral boundary conditions are determined from the basin-wide model, using an one-way nesting method. The external forcing to drive JCOPE2 includes wind stresses and net heat/freshwater fluxes at the sea surface converted from the six-hourly atmospheric reanalysis produced by the National Centers for Environmental Prediction/National Center for Atmospheric Research. Satellite sea surface temperature, sea surface height, and in situ temperature and salinity data were assimilated into the model based on a three-dimensional variational method [52]. The daily JCOPE2 reanalysis fields cover the period from January 1993 to the present. Comparison of simulated trajectories of passive particles carried by JCOPE2 and observed trajectories was performed in a previous study [20], which showed a satisfactory performance of JCOPE2 in simulating the three dimensional circulation across the western North Pacific Ocean.

### Particle-tracking scheme

A particle-tracking method was used to simulate the movement of v-larvae. The particles were carried by ocean currents in addition to having their own swimming behavior. The ocean reanalysis data of JCOPE2 served as the background ocean current. The specific v-larvae swimming behavior was then introduced into the experimental setup that was based on the particle-tracking scheme developed by Ohashi and Sheng [53]. The tracking scheme was based on the fourth-order Runge–Kutta method [54] with a tracking time step of three hours. The same tracking scheme was used previously by Chang et al. [20, 38, 55] for investigating the migration of Japanese eel larvae and adults in the western Pacific Ocean and was also used in simulations of the long-distance migration of adult American eels in the Atlantic Ocean [56, 57].

A random walk displacement was included in all experiments to represent unresolved sub-grid turbulent flow and other local processes [53]. The estimated maximum horizontal and vertical displacements due to the random walk were 600 m and 20 m during the simulation period, respectively. The duration of day and night were determined by the time of sunrise and sunset, which were set seasonally. Day length in spring and autumn was set to 12 hours (6 am to 6 pm) and was shortened to 10 hours (7 am to 5 pm) in winter (December–February), and lengthened to 14 hours (5 am to 7 pm) in summer (June–August).

#### Experimental design

Numerical experiments were conducted to examine the effect of larval swimming on larval transport routes and the recruitment success of the Japanese eel. The swimming behaviors considered in this study were diel vertical migration (DVM) and horizontal directional swimming in a particular compass direction. The migration experiments from the spawning area to East Asia (total migration, 290 days of transport, Fig 1) were first conducted to examine the potential effect of swimming behaviors in a general view. In order to better understand the detailed dynamics, the total migration was split into different scenarios geographically according to the potential influence of the NEC, STCC eddies, and the Kuroshio. The three scenarios were stage A: early transport from the spawning area, stage B: transport from the STCC eddy region, and stage C, transport from the southern Kuroshio. In each of the 3 specific stages, four experiments were examined, which were with/without DVM, with/without horizontal swimming (Table 1). This strategy of separating the migration into separate stages enables the effect of swimming to be clearly evaluated at each geographic area and life history stage of the migration.

**Table 1:** List of larval transport modelling simulation experiments to evaluate the effect of swimming by v-larvae at different times and locations of the larval migration of the Japanese eel, *Anguilla japonica*. Some v-larvae used swimming or performed diel vertical migration (DVM) and others did not as outlined in the text. Sensitivity simulations were also conducted using the conditions of DVM and the Total migraion experiments (see Discussion).

		Total migration				Stage A				Stage B				Stage C			
Eel life-history stage		preleptocephali →glass eel				preleptocephali → leptocephali				leptocephali				leptocephali → glass eel			
Duration (days)		290				160				70				60			
Release location		141–143°E/12-15°N (spawning area)				141–143°E/12-15°N (spawning area)				125–132°E/19-25°N (STCC eddy zone)				121–123°E/21.5–22.5°N (Kuroshio)			
Release date		1 May, 1 Jun, 1 Jul				1 May, 1 Jun, 1 Jul				1 Sep, 1 Oct, 1 Nov				1 Dec, 1 Jan, 1 Feb			
Exp. name		S1	S2	S3	S4	A1	A2	A3	A4	B1	B2	B3	B4	C1	C2	C3	C4
DVM		N	Y	N	Y	N	Y	N	Y	N	Y	N	Y	N	Y	N	Y
Swimming		N	N	Y	Y	N	N	Y	Y	N	N	Y	Y	N	N	Y	Y
If DVM	Day	50–340 m				50–210 m				210–280 m				280–340 m			
	Night	50–20 m				50–32 m				32–22 m				22–20 m			
If no DVM		150 m				150 m				150 m				150 m			
If swim	Speed	0−15 cm/s				0−12 cm/s				12−15 cm/s				15 cm/s			
	Direction	random/westward northwestward				northwestward				northwestward				northwestward			

The release region and time of v-larvae were chosen based on observations [28, 29]. In the total migration from the spawning area experiment, v-larvae were released within the spawning area from 141 to 143°E and 12 to 15°N with a separation distance of 10 km in both zonal and meridional directions (Fig 1). The release times were the 1^st^ day of May, June, and July. There were 1,800 v-larvae released each year. The tracking period was 290 days, considering the end of the main recruitment season between February to April. Following a previous study [20], horizontal swimming and DVM were set to be age-dependent and assuming a linear increase of body length. Swimming speed was set to increase from zero at release by 0.075 cm/s per day, with a maximum speed of 15 cm/s that would be reached when the larvae reach their maximum size [58]. As 24 hours continuous swimming assumed in previous studies [20, 38, 55] would be energy consuming during long-term migration and would not be compatible with using time for feeding, v-larvae were set to swim only during daytime and passively drift by ocean currents at night. DVM started on day 0 and got deeper linearly with the age of v-larvae from 50 m to 340 m during the day, and became shallower from 50 m to 20 m at night. If the larvae moved over bottom depths that are shallower than the diving depth, they were set to stay at 10% of water depth above the sea floor (i.e., stay at 90 m when water depth is 100 m). If there was no DVM, a fixed depth of 150 m was chosen where eel eggs were observed [25] and it would be an intermediate depth between the shallowest and deepest possible swimming depths during day and night throughout the migration.

With the objective of testing the hypothesis that active swimming in a direction towards the recruitment areas might increase recruitment success, three different swimming directions were examined in comparison to no swimming for the total migration simulations: random, westward, northwestward. The swimming directions were chosen according to what compass directions of swimming might help the larvae reach their recruitment areas based on the location of the spawning area in relation to the recruitment areas and the patterns of major currents in the western North Pacific. Therefore, we initially tested westward swimming (useful within the NEC) and northwestward swimming (the general direction of East Asia where the species lives) in the total migration experiment. In the random swimming scenario, the swimming speed also linearly increased with time, but the swimming direction was chosen randomly every 3 hours. The along current swimming strategy was not examined in present study because that strategy failed to reproduce v-larvae arrival into the inner shelf of the East China Sea [20].

In the experiments for stages A, B and C, settings of swimming speed and DVM followed the total migration experiment (Table 1), and the swimming direction was set to be northwestward. In stage A, release locations, times and number of v-larvae released were the same as the total migration experiment. The tracking period was 160 days. In stage B, the release area was in the STCC eddy region, extending from 125 to 132°E and from 19 to 25°N with the same spatial interval of 10 km (Fig 1). V-larvae were released on the 1^st^ day of September, October, and November according to the period when eel larvae were collected in this area [29]. There were 12,600 v-larvae released each year. The tracking period was set at 70 days. In stage C, v-larvae were released in the Kuroshio southeast of Taiwan, covering 121 to 123°E and from 21.5 to 22.5°N. The release times were chosen as the 1^st^ day of December, January, and February based on the observed eel larvae and glass eels in the Kuroshio and East China Sea [29]. There were 600 v-larvae released per year. The tracking period was 60 days.

**Fig 3:**
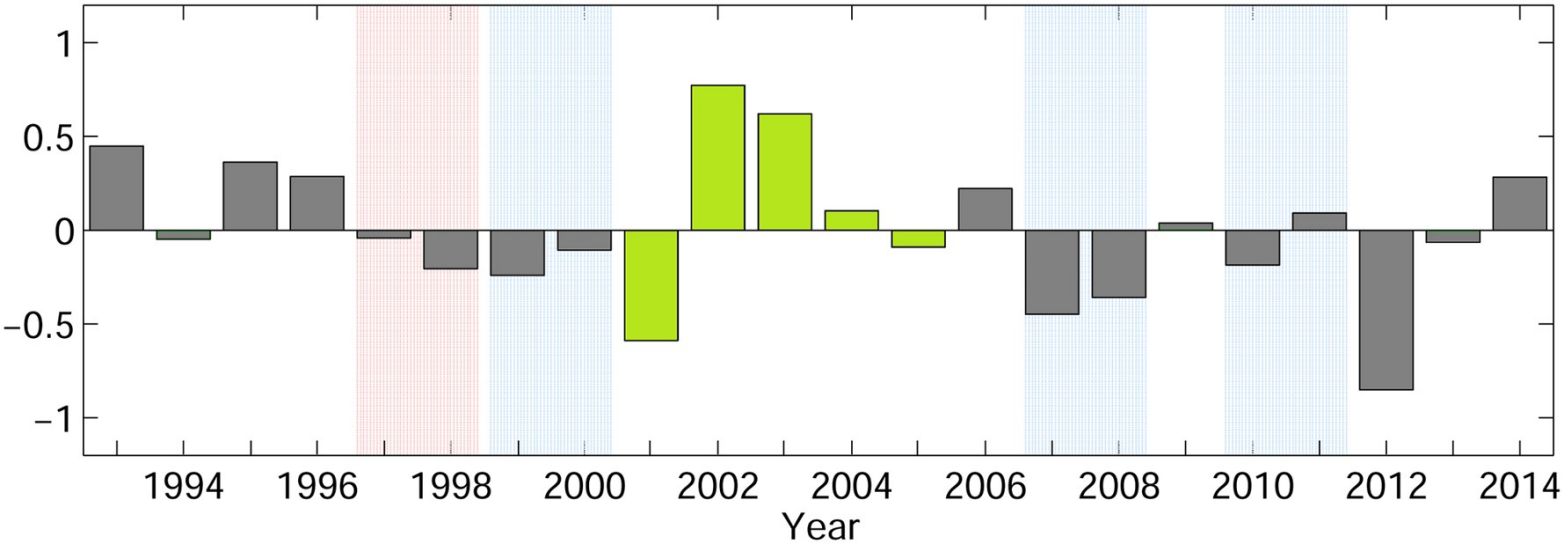
Annual Philippines-Taiwan Oscillation (PTO) index from 1993 to 2014. The annual PTO averages were taken from May to the next February, covering the simulation period. Red and Blue shading denotes extreme El Niño and La Niña events, respectively. Green bars are the 5 years chosen for simulation in the present study.

The selection of simulation years was using 5 consecutive years that did not include any extreme El Niño or La Niña event years. This resulted in the selection of 2001 to 2005 (Fig 3) that included various different states of the oceanic climate index called Philippines-Taiwan Oscillation (PTO) [59], which influences the western Pacific oceanic conditions, such as NEC bifurcation, the Kuroshio, STCC eddies, and can affect the shoreward migration pathways of Japanese eel larvae [20]. El Niño also has the potential to influence Japanese eel transport, but its actual role is not yet clear [18, 19, 60, 61] and appears to mostly be based on changes in the latitude of spawning [62]. Extreme El Niño and La Niña years were excluded, so these years consisted of a transition from low-negative to predominantly low-positive ENSO index values, and a range of NEC bifurcation values [63]. It can be noted though, that our objective of comparing larval directional swimming to no swimming could be achieved using any set of years, and we have also tested other years, which indicated the results were not sensitive to the years chosen.

The STCC eddy zone was defined as the area of 125°E to 150°E and 16°N to 25°N, and eddies in this area were identified using the Okubo−Weiss method [64, 65]. The arrival to the Kuroshio was defined when v-larvae entered the jet where the surface speed was greater than 20 cm/s. Reaching the ECS was defined when v-larvae cross the Kuroshio and arrived at the ECS shelf where water depth was shallower than 200 m. Significant differences in the arrival rates and times of reaching the defined locations were assessed using chi-square tests.

## Results

### Total migration: From spawning area to East Asia

**Fig 4:**
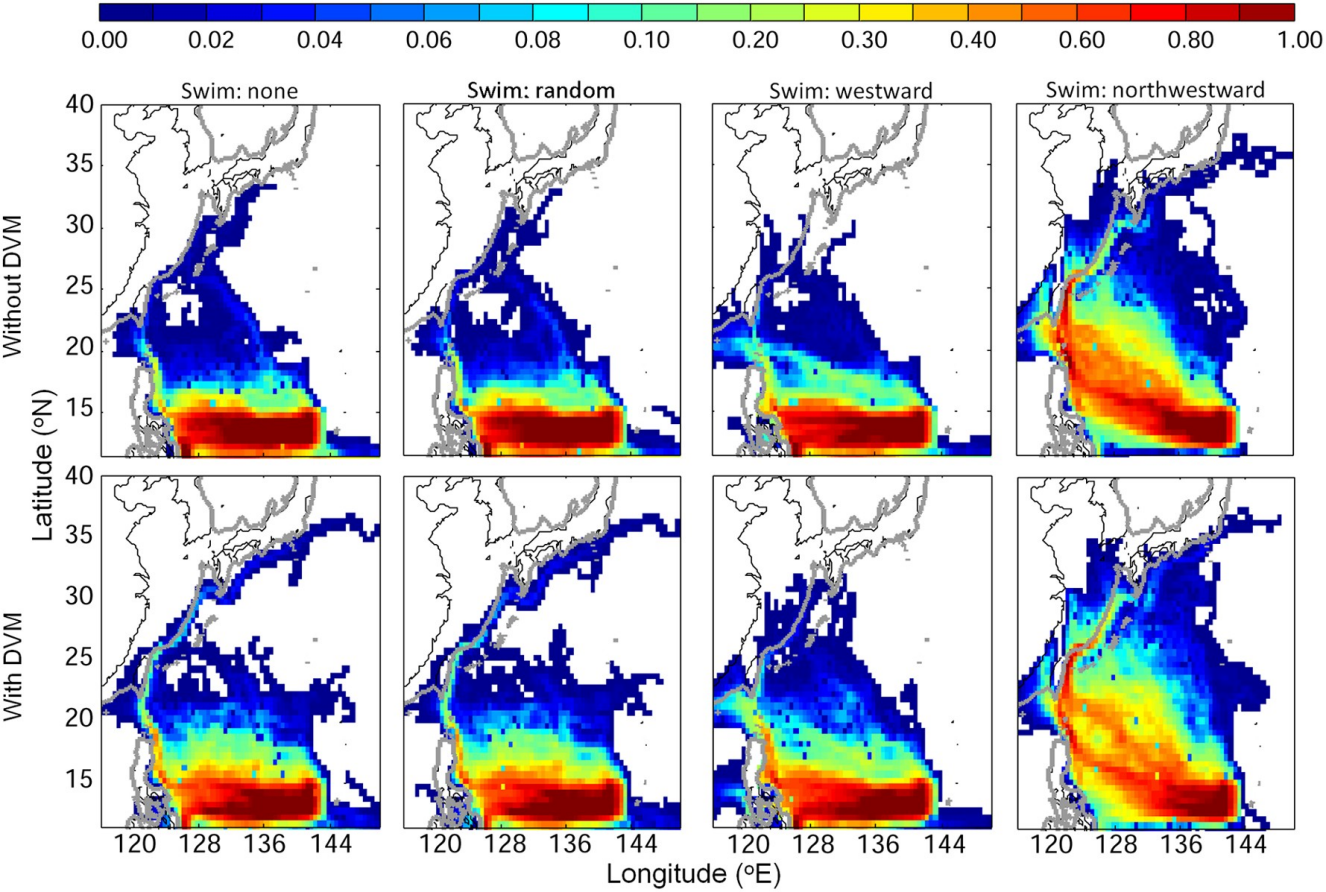
Visitation frequency distributions for the total migration experiment with different swimming strategies of (top) without and (bottom) with DVM. Swimming direction from left to right panels was non-swimming, random, westward, and northwestward swimming. V-larvae were released on the 1^st^ of May, June, and July in the spawning area, and were tracked for 290 days. The unit of visitation frequency was normalized by the number of v-larvae.

The dispersal patterns of v-larvae during the 290 days of the experiment showed some interesting differences among the 8 different swimming and DVM strategies (Fig 4). If v-larvae did not swim, they mostly were limited to south of 20°N, with only a few (~1%) v-larvae being able to each the East China Sea or near the south coast of Japan. Random swimming v-larvae had similarly limited northward dispersal towards East Asia, but some larvae were transported farther in the Kuroshio extension when they used DVM. The westward swimming v-larvae also showed little northward distribution. Instead, most of those v-larvae reached the east coast of Philippines and some of them continued moving westward through the inland seas of the Philippines or the Luzon Strait and into the South China Sea. A few westward swimming v-larvae were transported northward by the Kuroshio and moved over the continental shelf in the East China Sea. The northwestward swimming v-larvae showed a different general trajectory with many becoming widely distributed in the STCC eddy zone. Large numbers moved in a northwestward direction with some entering the Taiwan Strait, many entering the Kuroshio and then moving into the East China Sea, and some reaching the area along southern Japan. Fewer of the northwestward swimming v-larvae were entrained into the southward flow of the Mindanao Current near the Philippines compared to the 3 other swimming conditions. V-larvae with DVM showed wider dispersal than those without DVM. They would experience stronger ocean currents in shallow water during nighttime, leading to greater dispersal than without DVM.

Passive drifting or non-directional swimming resulted in very low arrival into the East China Sea beyond the edge of the Kuroshio (Fig 4). Among all cases, the northwestward swimming strategy produced dispersal patterns that were closest to the observed larval distribution pattern (Fig 1), including widespread movement into the East China Sea, frequent entrance into the STCC eddy zone, and entrainment into the Kuroshio. It also resulted in v-larvae reaching a wider region outside of the expected normal migration route at latitudes north of the spawning area. For successful recruitment to East Asia, the directional swimming was the better strategy, so the northwestward (NW, hereafter) heading was used in the following individual stage experiments.

### Stage A: Early larval dispersal

**Fig 5:**
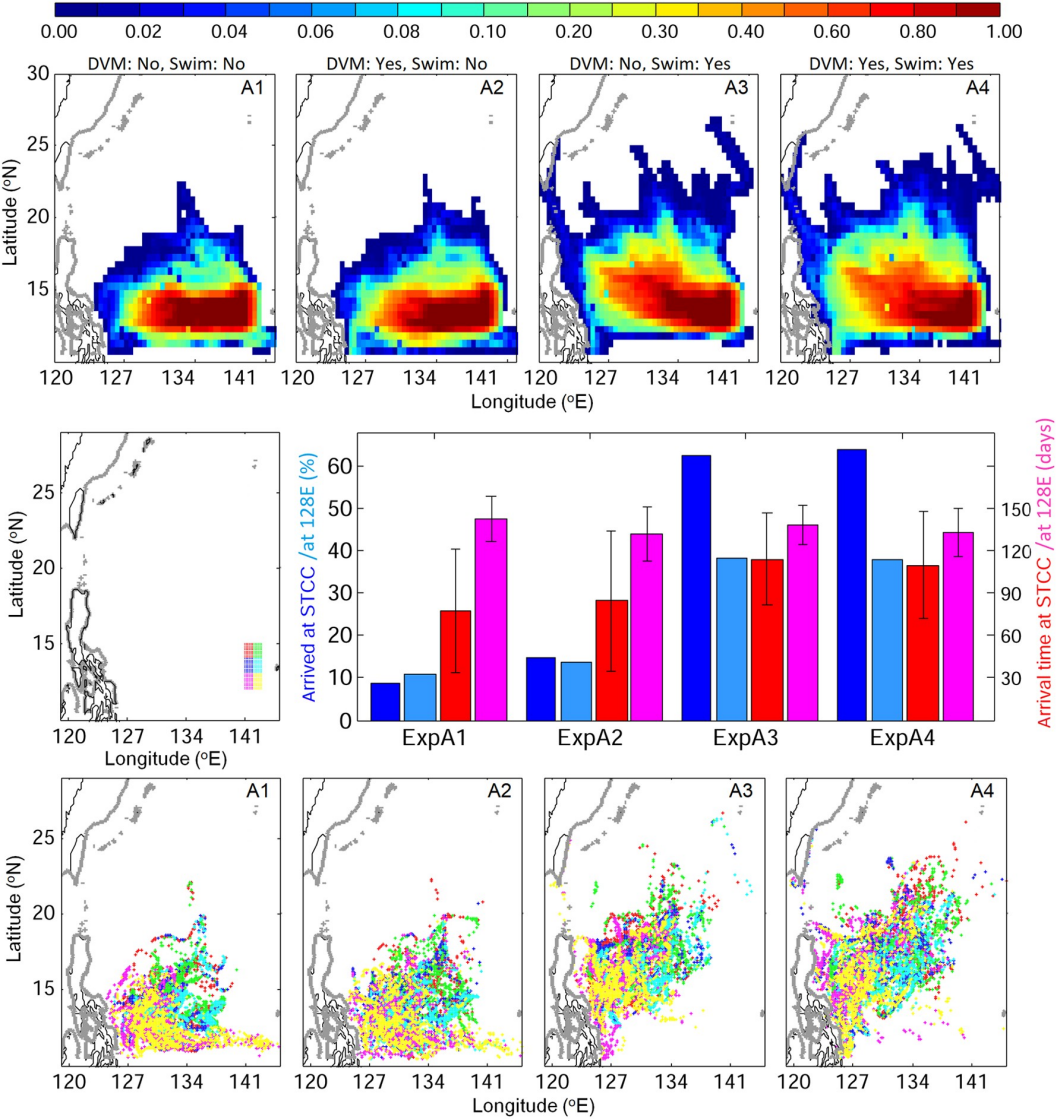
Visitation frequency for v-larvae in the early migration period (stage A) using different swimming behaviors (top), their arrival rates (left labels) and times (right labels) to the STCC eddy zone and 128°E (middle; bar colors correspond to y-axis label colors), and their final positions (bottom) categorized by their origins within the release area (middle left) coded with the different colors of their release areas.

For the 160-day simulation, v-larvae dispersion from the spawning area was evaluated for reaching either near the east coast of the Philippines at 128°E or the STCC eddy area. Non-swimming v-larvae mostly remained south of 18°N, being mainly between 11 to 17°N, and 10−15% of those v-larvae reached 128°E and about 10−15% entered the STCC eddy zone (Fig 5). Directional NW swimming v-larvae became distributed farther northward towards the STCC eddy zone and to the east coast of Philippines, from 12 to 22°N. About 60% of those v-larvae arrived at the STCC eddy region, and approximately 40% reached 128°E. Although significantly more reached the two areas with swimming (p<0.01), the time it took arrive there was less different (p>0.1). Northwestward swimming v-larvae also showed a similar distribution to the distribution of collected larvae (Figs 1 and 5), in which v-larvae appeared in both the NEC and STCC eddy area. In contrast, most no-swimming v-larvae did not enter the STCC area. V-larvae having DVM showed larger dispersal and had a better chance of arrival to the Kuroshio than those without DVM.

The final locations of v-larvae plotted by their release locations (Fig 5, bottom panels) showed that with no swimming, those originating from the southern spawning area ended up closer to the east coast of the Philippines or were transported southward or even eastward. Those originating from the northern spawning area were more likely to move northward towards the STCC eddy zone. Those using northwestward swimming became more mixed.

**Fig 6:**
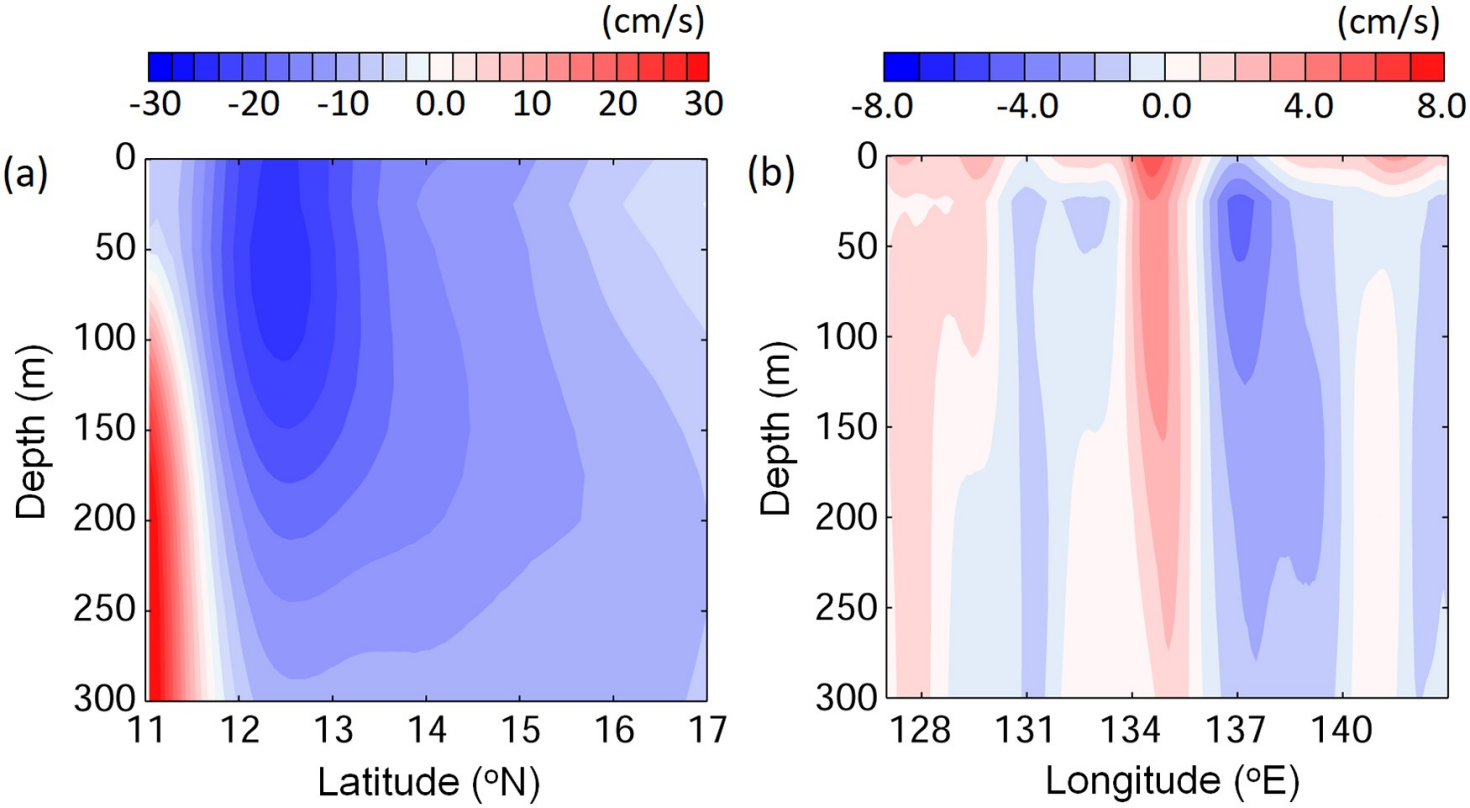
The vertical profiles of zonal (east-west) (a) and meridional (north-south) (b) velocities in the North Equatorial Current region. (a) was averaged from 125°E to 140°E, and (b) from 11°N to 17°N during the simulation period of stage A.

Evaluation of the ocean current to the west of the spawning area, showed that v-larvae that departed from the spawning area were mainly influenced by the NEC. During the simulation periods, the NEC was located from south of 11°N to 17°N and from the surface to a depth of > 300 m (Fig 6). The spawning region set in the present work was entirely within the NEC. The southern spawning area (12−13°N) was located near the core of the NEC (~20 cm/sec; Fig 6a), so v-larvae that departed from southern spawning area were transported by the relatively strong westward current and could arrive at the east coast of Philippines in a shorter time (Fig 5). In contrast, v-larvae that originated from the northern spawning area (14−15°N) were located in the northern NEC where westward velocity was relatively weak (~10 cm/sec). Meridional velocity (north or south) also existed in NEC, with a magnitude up to about 5 cm/s in some places (20−30% of zonal velocity). As a result, v-larvae such as from northern spawning would have been transported westward slower, and some would be advected northward into the STCC eddy zone. The highest average northward velocities occurred near 134−136°E, and this seemed to be reflected in the northward transport trajectories of v-larvae in Fig 5.

### Stage B: From STCC eddy zone to the Kuroshio

**Fig 7:**
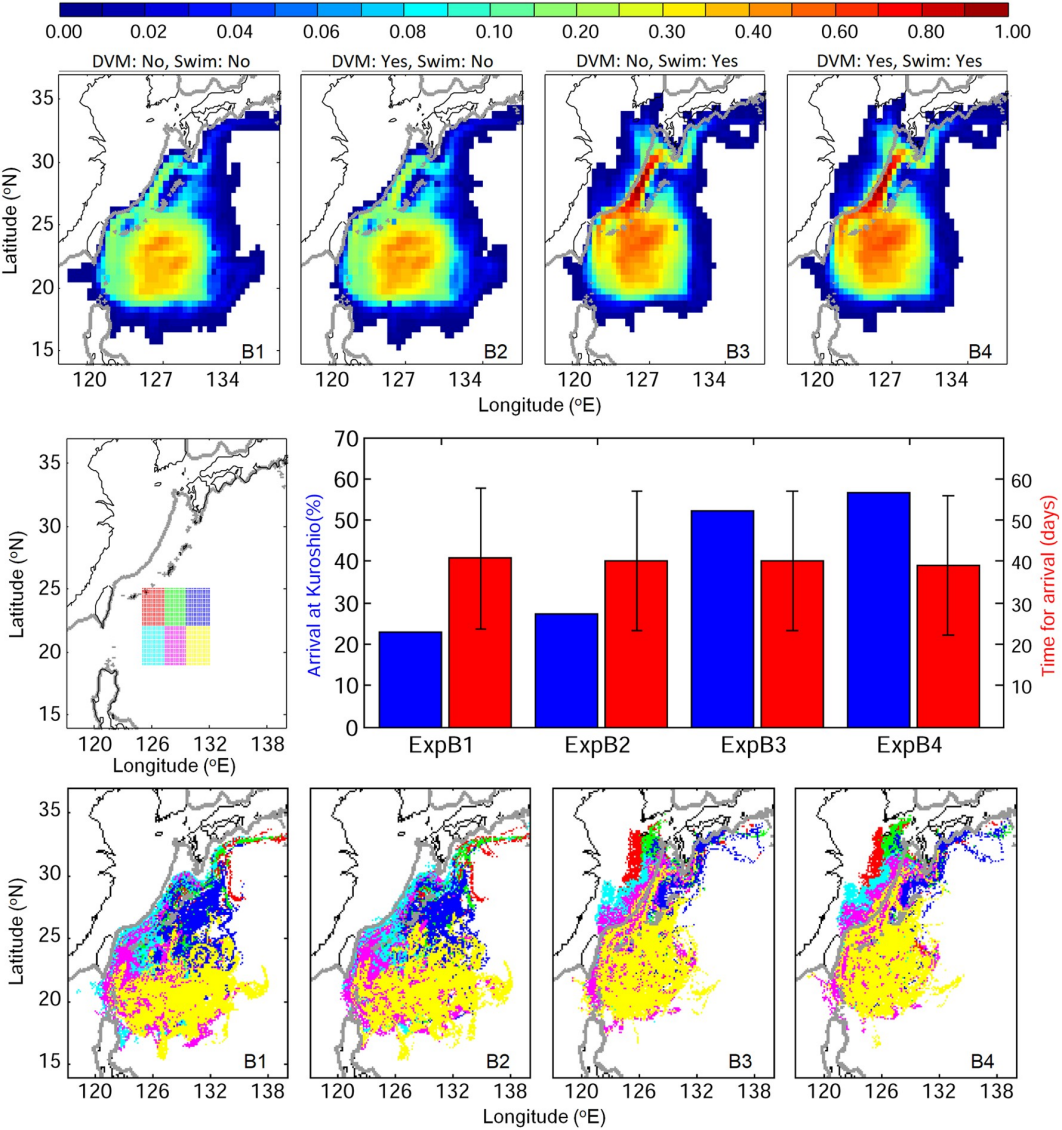
Visitation frequency for v-larvae released in the Subtropical Countercurrent (STCC) eddy region (stage B) using different swimming behaviors (top), their arrival rates (left labels) and times (right labels) to the Kuroshio (middle), and their final positions (bottom) categorized by their origins within the release area (middle left) coded with the different colors of their release areas.

V-larvae released in the STCC eddy zone were evaluated for reaching the Kuroshio for successful recruitment potential within the 70-day simulation period. Non-swimming v-larvae showed greater dispersal to the east and northeast from the STCC eddy zone than the cases with swimming ability (Fig 7). Dispersal of non-swimming v-larvae extended east of the release region to 135°E, whereas swimming v-larvae were mostly distributed west of 132°E. Swimming v-larvae had greater arrival rates to the Kuroshio, as well as to the East China Sea shelf. The swimming behavior increased the arrival rate to the Kuroshio and East China Sea by about 30%. Some entered the southern Kuroshio, and many moved directly to the northwest. Some also crossed over and moved far into the East China Sea, whereas few v-larvae without swimming moved away from the western or northern edge of the Kuroshio. With or without DVM had a minor influence on their dispersal, but DVM could slightly enhance the arrival rate to the Kuroshio by 3−5%. The number of swimming v-larvae arriving at the Kuroshio was higher (56.8% and 52.4% with swimming compared to 27.1% and 22.9% for non-swimming, p<0.01), but the time it took was similar (~40 days, p>0.1).

**Fig 8:**
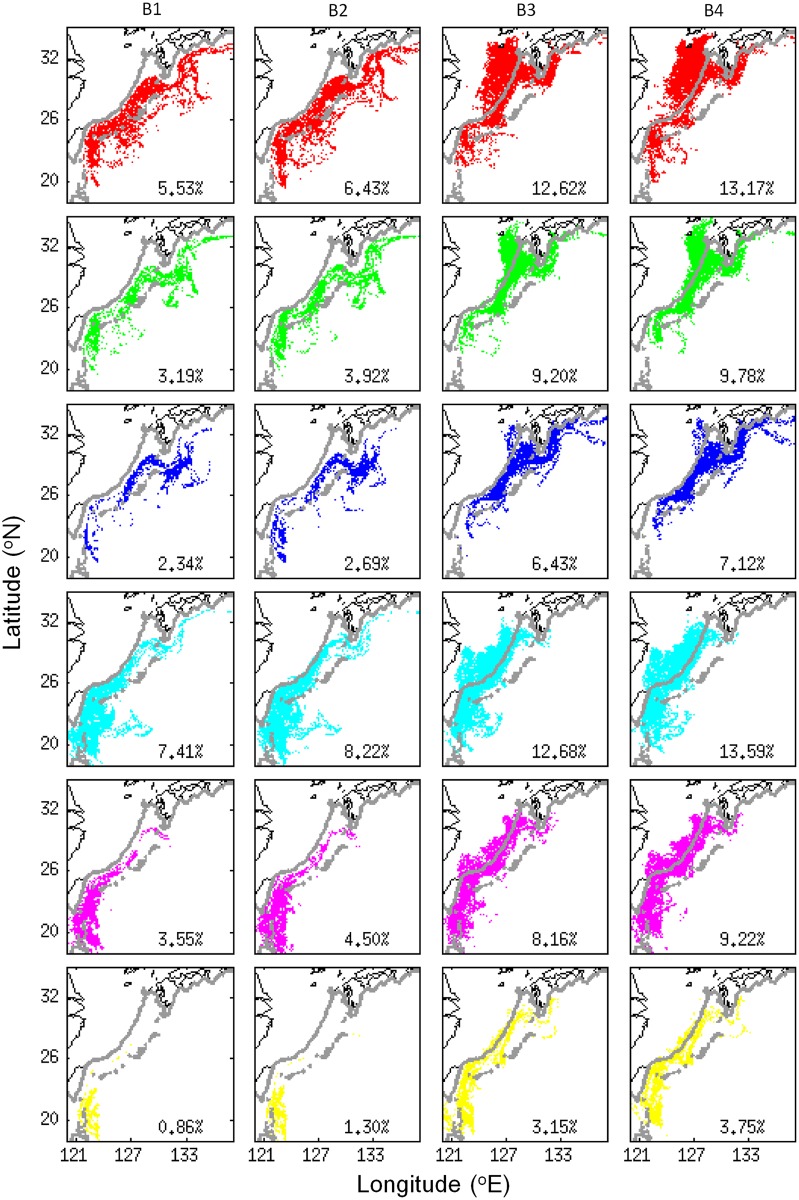
Final positions for the v-larvae that successfully arrived at the Kuroshio separated by their release locations in the STCC region. The colors and rows of panels correspond to the release location colors in the middle left panel in Fig 7, and the columns of panels correspond to the types of swimming behaviors in experiments B1-4 in Fig 7. The numbers show the percentage of arrival to the Kuroshio.

V-larvae originating from the northwestern STCC region were likely to approach the Kuroshio and East China Sea within the simulation period. In contrast, those originating from the southeastern STCC area mostly remained in the eddy zone to the end of the simulation, especially with no swimming (Fig 7). Tracing v-larvae that had entered the Kuroshio, part of the non-swimming v-larvae were still able to leave the eddy zone and reached the Kuroshio and continued on within the current to the south of Japan (Fig 8). V-larvae having swimming behavior showed greater arrival rates to the Kuroshio from all the release areas. Moreover, part of them could even cross the Kuroshio and reach the East China Sea shelf, suggesting the effectiveness of the swimming strategy for recruitment success. Those that entered different parts of the East China Sea showed some clear patterns according to their release locations, such as those from the southwest release area entering the southern and central parts of the East China Sea (Figs 7 and 8). Other v-larvae were transported directly northward and may have entered the Kuroshio south of Japan. It was also apparent that some v-larvae that initially entered the Kuroshio could later be entrained out of the current (eastward) by eddies and end up offshore again, especially in the non-swimming cases (Fig 8).

**Fig 9:**
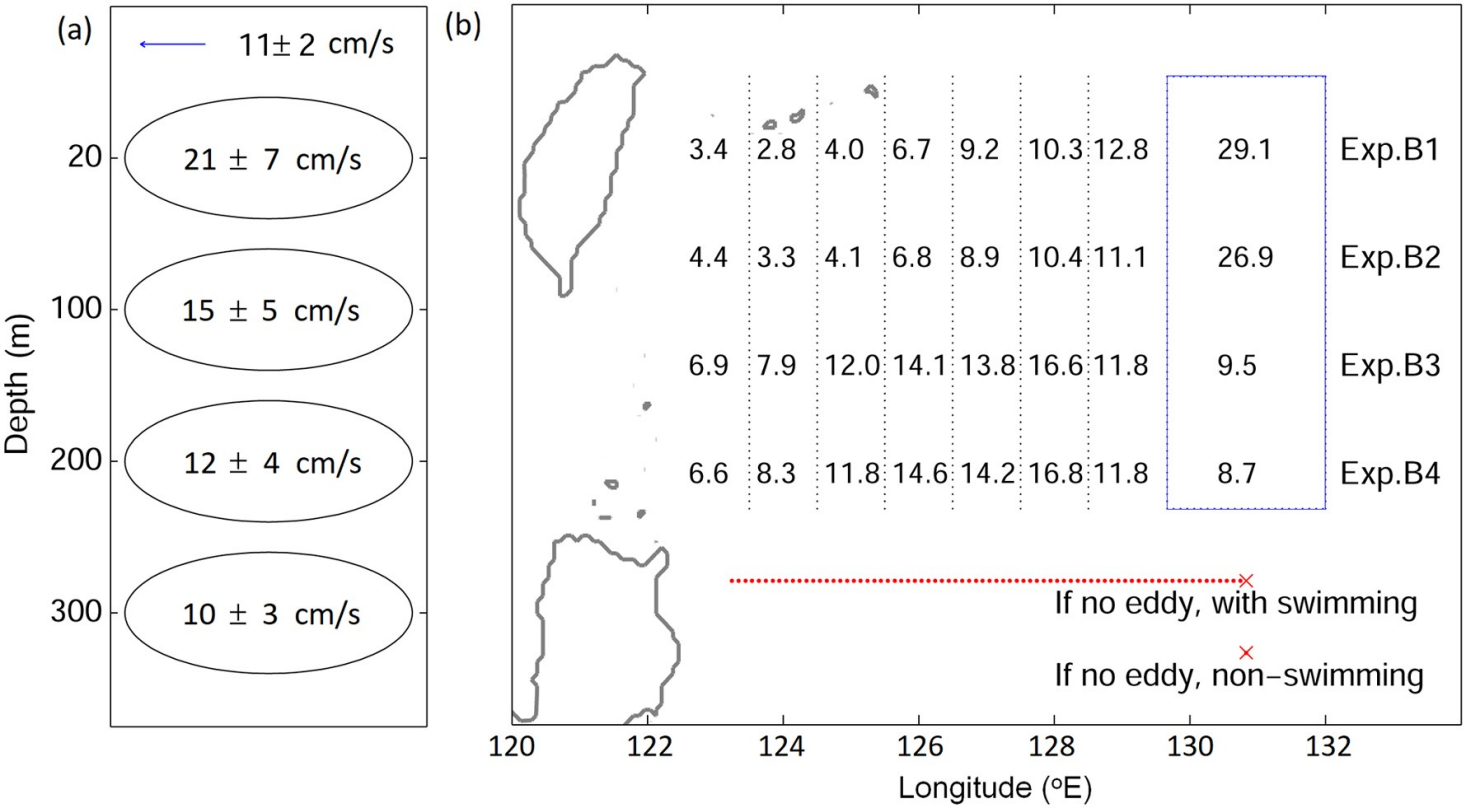
Mean eddy rotation speeds at various depths in the STCC eddy zone (125°E to 132°E and 19°N to 25°N) during stage B (a). Percentage of v-larvae at different longitudes (pooled from 19°N to 25°N, black dotted lines) on day 70 after being released from the blue box (eastern release area in stage B) for non-swimming v-larvae (Exp. B1 and B2) and swimming v-larvae (Exp. B3 and B4). Examples of the distance that could be traveled if there was swimming with no ocean currents or eddies is shown with a red cross (starting point) and line in the bottom of the map.

Despite being relatively close to the Kuroshio, < 30% of non-swimming v-larvae could reach the Kuroshio. The positions of STCC eddies was likely the major factor affecting the v-larvae in this region because the 200−300 km wide mesoscale eddies would have been widely distributed there (Fig 2). Only a few v-larvae could avoid entering eddies in the STCC area because more than 90% of the v-larvae had encountered eddies at least once during the 70-day simulation. During the simulation period, the mean rotation speed *U* within eddy interior was 10−20 cm/s and the mean propagation speed *c* was about 10 cm/s, leading to the nonlinearity *U/c* of 1−2, suggesting v-larvae would likely have been trapped by eddies (Fig 9a). However, some non-swimming v-larvae trapped in these eddies must actually be transported out of the area and into the Kuroshio because more than 20% of non-swimming v-larvae reached there. If there is no ocean current or eddies, non-swimming v-larvae would simply stay at the released point (Fig 9b, bottom cross mark). Looking at the percentages of larvae that were released at the eastern two areas in the STCC zone, nearly 50% of non-swimming v-larvae were transported 100−800 km westward by eddies (Fig 9b, Exp. B1 and B2), indicating eddies help to transport non-swimming v-larvae. However, eddies also reduce the ability of the swimming v-larvae to reach the Kuroshio, because larvae swimming from 131°E with no currents or eddies would be able to migrate 900 km and arrive at the edge of Kuroshio around 123°E (Fig 9b, red dotted line). In the simulation, more than 80% of swimming v-larvae were distributed east of 123°E (Fig 9b, Exp. B3 and B4), suggesting the swimming v-larvae were decelerated by eddies.

### Stage C: From the Kuroshio to East Asia

**Fig 10:**
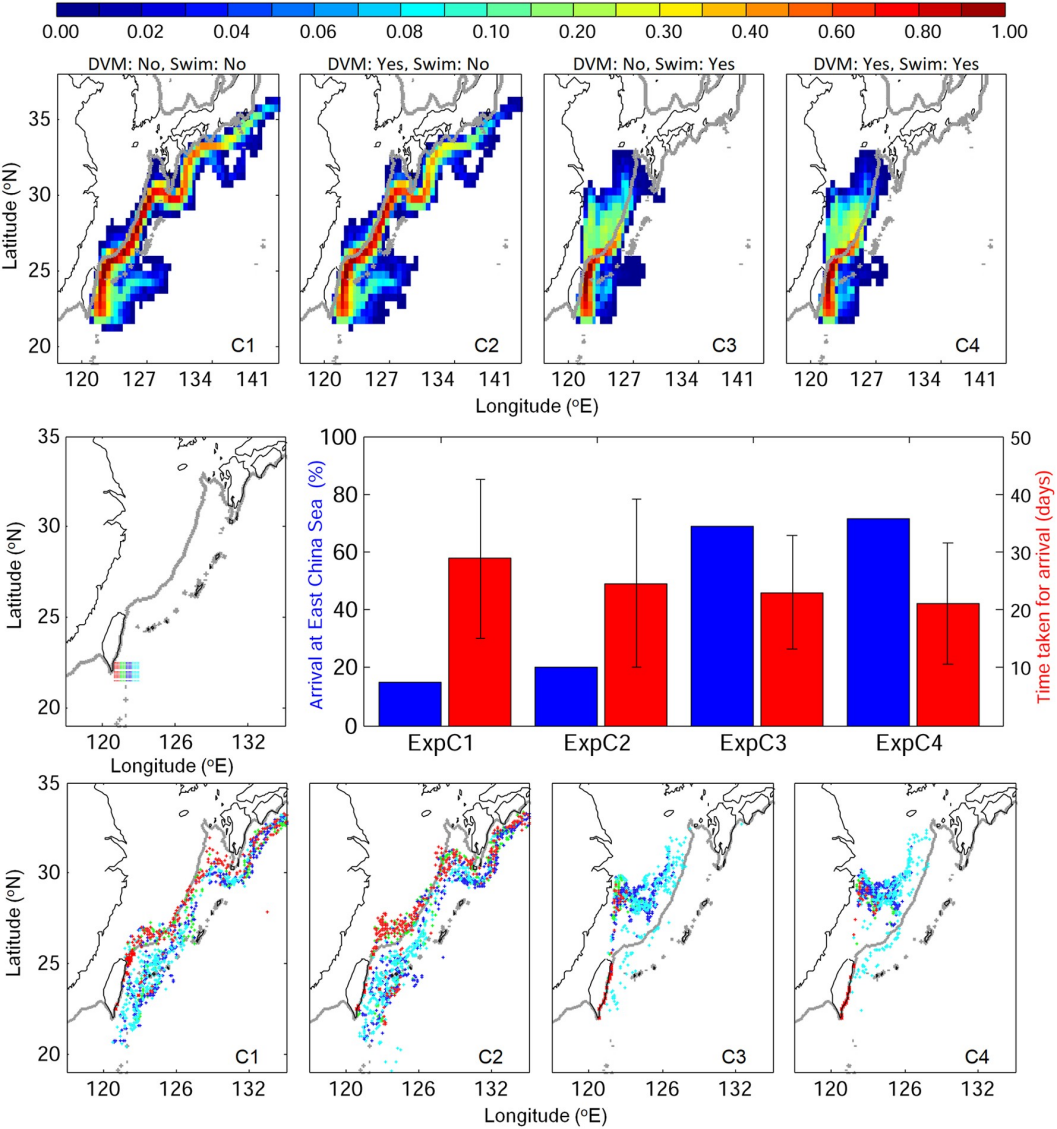
Visitation frequency for v-larvae released in the southern Kuroshio (stage C) using different swimming behaviors (top), their arrival rates (left labels) and times (right labels) to the East China Sea (middle), and their final positions (bottom) categorized by their origins within the release area (middle left) coded with the different colors of their release areas.

The v-larvae released within the Kuroshio southeast of Taiwan were evaluated for successful recruitment to the East China Sea shelf or to the south coast of Japan during 60 days. Non-swimming v-larvae were mostly distributed in the Kuroshio, from east of Taiwan to south of Japan, but some crossed over onto the shelf in the southern East China Sea (Fig 10). However, numerous swimming behavior v-larvae could make it to eastern Taiwan or into the East China Sea and move over the continental shelf. Only a few stayed in the Kuroshio long enough to arrive at the south coast of Japan. About 20% reached the east coast of Taiwan. Considering the arrival to East China Sea shelf, 15−20% of non-swimming v-larvae arrived at the outer shelf along the western edge of the Kuroshio, and swimming v-larvae had higher arrival rate of 70−75% and reached quite far onto the shelf. The time it took to reach the East China Sea was slightly shorter (p = 0.2 without DVM and p = 0.5 with DVM) with swimming ability. With or without DVM did not influence the dispersal significantly, however, it could shorten the duration to the East China Sea shelf by a few days, especially in the non-swimming condition. The final distribution of non-swimming v-larvae mostly remained in or near the Kuroshio, but a few of them that originated from the western edge of Kuroshio were able to reach the edge of the East China Sea shelf. Some non-swimming v-larvae that entered the East China Sea would again be carried off the shelf by the Kuroshio. Those released on the east side of the Kuroshio mostly showed quite different final locations that were east or south of those released on the west side. The swimming v-larvae that originated from the center and eastern side of Kuroshio mostly entered into the East China Sea shelf, and some from the east side were the only swimming larvae not to recruit successfully. Those originating from western Kuroshio mostly reached the east coast of Taiwan, but some reached the western East China Sea.

**Fig 11:**
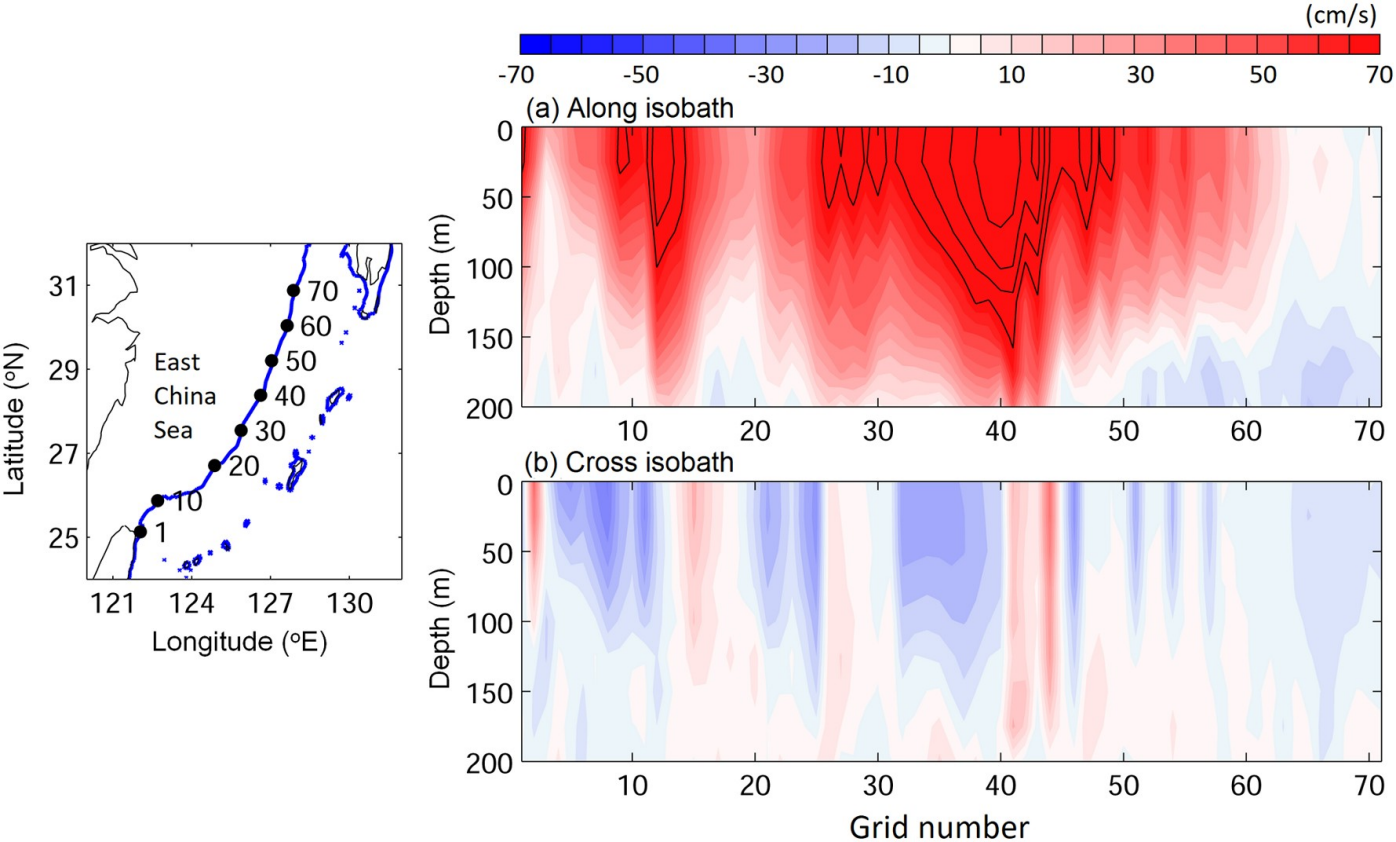
Vertical profile of ocean current along (a) and across (b) isobaths along the 200 m isobath in the East China Sea. The left map panel shows the grid numbers along the 200 m isobath (blue line), which correspond to x-axis numbers in the right panels. Positive values indicate flow to the northeast and offshore (to the east) for along and across the isobath, respectively.

Analysis of the northeastern along-axis flow of the Kuroshio over the 200 m isobath along the edge of the continental shelf in the East China Sea showed that there was significant flow over the slope at depths of 200 m (red in Fig 11a). There was also onshore flow (blue in Fig 11b) in the areas where non-swimming v-larvae crossed over the 200 m contour in Fig 10, indicating that most of the larvae considered to have successfully recruited were likely still within the flow of the Kuroshio, or were cast off slightly onto the continental shelf by onshore flows.

## Discussion

The larval transport modelling strategy of the present study and the examinations of the ocean currents of the NEC and the Kuroshio east of Taiwan, along the continental shelf in the East China Sea, and in the STCC eddy region, provided some clear information about the effect of directional swimming on the larval migration of the Japanese eel. The unusually long larval duration of eel larvae makes them susceptible to large-scale dispersal by ocean currents or to become trapped in eddy regions, so the question of whether or not these larvae can use directional swimming to facilitate successful recruitment has remained a key question for understanding leptocephalus ecology [2, 15, 43, 66–68]. Leptocephali clearly have substantial swimming abilities including for short or even long periods of time as seen in captivity and more rarely in the ocean [43, 51, 69–71], and our analysis shows that swimming in a NW direction towards their recruitment areas would likely be beneficial to increase the recruitment success of the Japanese eel.

For the total migration experiment in which larvae were released in the spawning area and were tracked for 290 days, the patterns of simulated v-larvae dispersion suggested that passive or random swimming were similar, but were not the most effective strategies. Some larvae could reach the East China Sea and south of Japan without using directional swimming, mostly by transport in the NEC and Kuroshio, but they were much fewer, especially compared to those that used NW swimming behavior. The larvae with NW swimming entered the northern South China Sea in greater numbers and moved farther into the East China Sea where they have not entered in previous modelling studies [18–21], except during a few simulation years in a recent study using an air-sea coupled model [62]. This suggests that the swimming speeds of the v-larvae that increased with age were enough to influence the movements of the larvae. For the random directional swimming condition of the v-larvae, the sum of all the randomly chosen directions would add up to be nearly zero, which would lead to ineffective swimming, and likely explains the lack of increased dispersion of those larvae except within the Kuroshio Extension when DVM was used. The random correspondence in timing of more along-current swimming while at the shallow nighttime DVM depths within the Kuroshio may account for this farther transport in the Kuroshio compared to that not occurring with no DVM.

**Fig 12:**
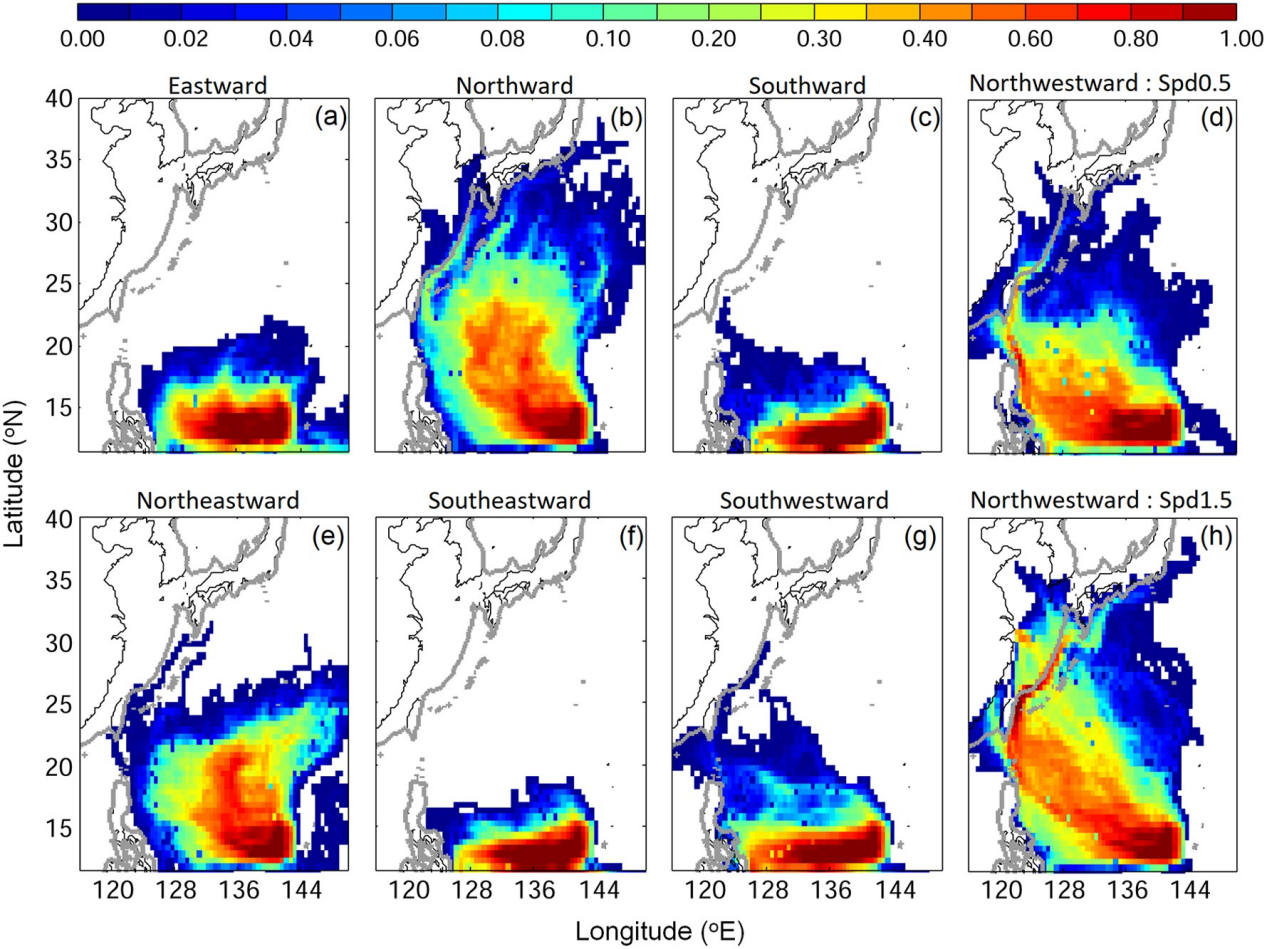
Same as Fig 4, but for different swimming strategies. Swimming towards east (a) north (b) south (c) northeast (e) southeast (f) southwest (g), and northwestward swimming but with 50% swimming speed (d), and 150% swimming speed (h). All cases performed DVM.

The NW swimming direction was chosen because it is the most logical direction for larvae to move from the spawning area towards their recruitment areas and to avoid southward entrainment into the Mindanao Current, which can increase greatly with more southern spawning locations [20, 62]. However, it is also important to know what effects other swimming directions have on larval movements, so we conducted a sensitivity experiment using various swimming directions to help evaluate the implications of the NW swimming. This showed that swimming away from the recruitment areas in eastward, southward, and southeastward directions resulted in little v-larvae arriving at the Kuroshio east of Taiwan (Fig 12). Northward swimming resulted in a wider distribution towards East Asia and could potentially be an option for entering Japanese rivers. However, v-larvae distribution in the STCC eddy zone was mostly concentrated further east of where larvae have been collected at higher latitudes (Fig 1; [49]) and the success to reach recruitment areas was lower than with NW swimming.

Our experiment examining the early larval transport from the spawning area for 160 days (stage A) found similar effects of the NW swimming, with more larvae reaching the area east of the Philippines, and even more entering the eddy region of the STCC. Many larvae tended to move at a northwestward angle compared to those with no swimming, which shows swimming affected their trajectories especially after about 100 days. Northward flow was evident at some longitudes in the average meridional velocity section, which might contribute to transport of larvae into the STCC eddy region. Few of the NW swimming larvae entered the southward flow of the Mindanao Current compared to the non-swimming larvae, because of the northward component of their swimming. This appears to indicate that NW swimming can reduce southward transport into the Mindanao Current such as during strong El Niño or Philippines−Taiwan Oscillation (PTO) years as seen in previous studies [18–20]. The 160-day period was not long enough for any larvae to reach the Kuroshio near the Philippines without swimming, except for some that used DVM and likely experienced stronger currents at shallow depths.

In the experiment about larval transport from the STCC eddy area for 70 days (stage B), at least a few non-swimming larvae could enter the Kuroshio through eddy transport, but higher percentages were successful when using NW swimming. In this region, the use of DVM increased the success rate compared to staying at a fixed intermediate depth (at 150 m), likely because of faster current speeds in shallower depths. Some larvae with both swimming or no swimming appeared to be transported north by the Ryukyu Current [72] and could have entered the Kuroshio south of Japan. Although westward propagating eddies allowed some non-swimming larvae to reach the Kuroshio, the eddies slowed down some swimming v-larvae that would have been able to reach the edge of the Kuroshio in an absence of eddies. The role of eddies depends on the relative speeds between eddy propagation and swimming of v-larvae [38]. Westward propagating eddies would accelerate slow-swimming v-larvae (slower than eddy propagation of ~10 cm/s) but would decelerate the fast-moving v-larvae (faster than eddy propagation) while they were inside the eddy. The abundance of these STCC eddies can fluctuate seasonally as well as inter-annually [32, 59, 73], and a previous transport modelling study proposed that the glass eel recruitment in Taiwan could be connected to the abundance of STCC eddies [38]. STCC eddies can modulate the strength of the Kuroshio [35], which can further influence the transport and arrival of eel larvae. However, a recent study suggested a weaker trapping ability of eddies based on rotationally coherent Lagrangian vortices [74]. Rypina et al [75, 76] found that the diffusivity was enhanced at the eddy periphery, which may speed up mixing and reduce eddy trapping ability.

The most unique experiment of the present study was the evaluation of the effect of swimming by larvae that were released at the origin of the Kuroshio and tracked for 60 days (stage C). This showed swimming had a clear effect to allow larvae to cross out of the strong current (70−75% crossing with swimming, < 20% with no swimming), which confirms previous suggestions that swimming likely needs to be used by larvae to be able to cross western boundary currents [43, 44]. Some non-swimming v-larvae crossed over onto the continental shelf, but some of that was likely due to Kuroshio intrusions onto the shelf, which are common in winter [57], and also because the strong flow of the current passes over depths < 200 m along the slope and can sometimes have a multi-core structure [77, 78]. Almost all of the NW swimming larvae released in the Kuroshio reached Taiwan or were able to cross out of the Kuroshio and enter far into the East China Sea with or without DVM, which illustrated the effectiveness of swimming to detrain from the current.

Therefore, the results of the 4 experiments we conducted all found that active NW swimming by the larvae could significantly affect the transport trajectories and the final locations of the larvae after the specific number of days. The sensitivity experiments also showed that other swimming directions except for northward were clearly not effective compared to no swimming. The passive swimming strategy with various forms of DVM was used by earlier studies on Japanese eel larval transport [18, 19, 21] and similar results were obtained as in our total migration route experiment. Those studies found extensive dispersal, with limited entry into the Kuroshio. This indicates that directional swimming is much more effective than the no-swimming conditions, which did not facilitate entering and crossing out of the Kuroshio.

Similar results showing extensive larval dispersal were obtained in the larval transport modelling studies on the Atlantic eels that spawn in overlapping areas of the Sargasso Sea and then recruit to either the west (American eel, *A*. *rostrata*) or east (European eel, *A*. *anguilla*) sides of the North Atlantic basin [68] using either no DVM [79–82] or DVM [18, 44, 46, 83, 84] behavior by the larvae. Active swimming was found to be more effective for simulated larval migration across the North Atlantic for the European eel [46]. In addition, the apparent disappearance (lack of collection) of European eel leptocephali that are larger than about 60 mm within the Sargasso Sea gyre [43, 68], despite apparently high retention of larvae in the spawning area region [45], has suggested that the large-size larvae may eventually begin swimming out of the gyre [43]. The American eel makes a similar larval migration as the Japanese eel in which it must enter and cross the western boundary current of the North Atlantic that is analogous with the Kuroshio, the Florida Current/Gulf Stream. Like in the Kuroshio region, the modelling study of Rypina et al. [44] found that very few larvae would reach the 200 m isobath of the continental shelf without using directional swimming to the west or NW. That study and our results suggest that oriented swimming could be beneficial for both the Atlantic eel species if they evolved 2 different directional swimming behaviors that are expressed in the larger larvae, such as NW swimming for the American eel and northeast swimming for the European eel.

The results of the ocean circulation model simulations of the present study seem to establish that it is at least theoretically possible that active directional swimming can facilitate larvae to move towards their recruitment areas, but the potential biological significance of these findings in the ocean is a more complex subject. Our v-larvae had swimming speeds of about 2 BL/sec up to 15 cm/sec by the time they reached large sizes, and the larvae swam throughout daylight hours. A laboratory study on the swimming-ability of anguillid glass eels and conger eel leptocephali did not analyze their sustained swimming speeds [69]. But direct measurements 9 species of late-stage coral reef larvae of much smaller sizes (< 25 mm) found they had sustained swimming speeds (for 24 hr) of about 8−25 cm/sec [85], so the speeds we used for leptocephali may be reasonable. To evaluate the effect of this choice of speed, we also tested the sensitivity of swimming speed for NW swimming using a 50% slower swimming speed (maximum 1 BL/s, Fig 12d) and a 50% faster swimming speed (maximum 3 BL/s, Fig 12h). The fast swimming showed greater movement into the East China Sea and more larvae reached China and Korea. Some of the slower swimming v-larvae were still able to reach Taiwan and move over the East China Sea shelf at greater rates than the no/random swimming v-larvae (Fig 4).

Artificially spawned and reared Japanese eel leptocephali were observed to be able to swim continuously for at least 2 months with no food, because their bodies contain large amounts of transparent energy storage compounds [43]. This suggests these larvae may be highly adapted for sustained swimming. There is also increasing awareness that other fish larvae may use active swimming to reach their recruitment habitats [86, 87]. Therefore, it seems possible that both anguillid and marine eel leptocephali may have the ability to use swimming to increase their recruitment success if ocean currents do not take them where they need to go.

Several other factors also need to be considered when evaluating the possible trade-offs and the likelihood of the evolution of directional swimming by eel larvae. For example, using sustained swimming when leptocephali are still small and are feeding and growing may not be adaptive. The energy expenditure of swimming would slow down early growth, and the lower swimming speeds of the small size larvae would make it less effective relative to the current speeds. However, for larger larvae that have mostly finished their growth, the use of directional swimming behavior seems as though it would be highly adaptive according to the findings of the present study. In particular, if larvae are transported out of the westward flow of the NEC and enter the STCC eddy region, directional swimming would help them escape that area and enter the Kuroshio more quickly. Then for the larvae within the Kuroshio, no mechanism has been identified that can take them across the current, which means active swimming would be required. This has been confirmed by the present study that showed swimming out of the current, and this and previous modelling studies found little or no detrainment from the Kuroshio or Gulf Stream in the Atlantic without swimming. However, the sub-grid scale processes such as turbulence that was not fully resolved in the model we used may also influence eel larvae migration. It would be useful to construct a high-resolution model that incorporates spatially and temporally dependent diffusivity in the random walk simulations to explore the role of turbulence.

Although the present study demonstrates that increasing daytime swimming in accordance with the growth of the larvae could be an effective strategy to increase recruitment, it is only a first view of this subject that can set the stage for further studies that consider more detailed biological or oceanographic factors. The cost/benefit of early swimming compared to swimming behavior only being used by the larger larvae in relation to the energetics of swimming and growth may provide other useful information. Determining what speeds of swimming and amount of time spent swimming are needed to facilitate successful movements in each area could also be explored. The orientation mechanism of larvae is also a future subject to be evaluated.

What our study suggests though, is that if eel larvae can have genetically programmed directional swimming behaviors, this could increase the number of larvae reaching their recruitment areas. Eels and other migratory marine animals can have a variety of sensory systems including a geomagnetic sense that are used to make remarkable migrations to specific areas [88, 89], so directional swimming by leptocephali seems to be a possibility. If leptocephali of species such as the Japanese eel have evolved this ability, it would likely tend to counteract the effects of ocean-atmosphere events or changes that reduce the effective transport of larvae by ocean currents. However, interannual fluctuations in recruitment occur in this and other anguillid species, and a recent study found that Gulf Stream variations could influence American eel larval recruitment [90]. It will be interesting to test the effect of directional swimming during other more anomalous years to help understand the larval migrations these interesting eel species that use the ocean for larval development and growth.
